# Within-player associations between GPS-derived external training load and heart rate-based internal training load in elite youth male soccer training

**DOI:** 10.1371/journal.pone.0357176

**Published:** 2026-09-11

**Authors:** Krisztián Havanecz, Tímea Téglás, Leonidas Petridis

**Affiliations:** 1 Training Theory and Methodology Research Center, Hungarian University of Sports Science, Budapest, Hungary; 2 Department of Sports Medicine and Digital Health, Faculty of Health and Sports Sciences, University of Győr, Győr, Hungary; 3 Scientific Research Centre of the Hungarian Defence Forces, Transformation Command, Budapest, Hungary; 4 Research Center for Sport Physiology, Hungarian University of Sports Science, Budapest, Hungary; Portugal Football School, Portuguese Football Federation, PORTUGAL

## Abstract

**Objectives:**

This study examined within-player associations between GPS-derived external training load variables and heart rate-based internal training load during in-season training in elite youth soccer players. A secondary aim was to identify which intensity-based external load variables are independently associated with heart rate (HR) exertion per minute.

**Methods:**

Eleven under-17 male soccer players were monitored across a nine-week in-season period. 266 player-session observations were included. External training load was assessed using GPS units with integrated inertial sensors, including total distance covered, high-speed running distance, explosive efforts, player load, and their time-normalized derivatives. Internal training load was quantified using heart rate exertion derived from individualized HR zones. Within-player associations were examined using repeated-measures correlations. A linear mixed-effects model was used to examine predictors of heart rate exertion per minute.

**Results:**

HR exertion showed nearly perfect within-player associations with total distance covered (r = 0.921, p < 0.001) and PlayerLoad^TM^ (r = 0.924, p < 0.001), while large associations were observed for high-speed running distance (r = 0.662, p < 0.001) and explosive efforts (r = 0.621, p < 0.001). Intensity-based variables, including total distance covered per minute and explosive efforts per minute, were significant positive predictors of HR exertion·min ^−^ ¹.

**Conclusions:**

In elite under-17 youth soccer training, cumulative HR exertion primarily reflects accumulated external training volume, whereas HR exertion·min ^−^ ¹ is more closely related to locomotor and explosive action intensity. These findings highlight the need to interpret cumulative and intensity-based internal training load metrics separately in within-player training monitoring.

## Introduction

Systematic monitoring of training load (TL) is a central component in modern elite soccer. TL is commonly conceptualized through the distinction between external training load (ETL) and internal training load (ITL) [1]. ETL describes the physical work performed by the athlete, whereas ITL reflects the individual psychophysiological response to that work [2, 3]. ETL is commonly derived from wearable tracking systems, including GPS/GNSS and inertial sensors, whereas heart rate (HR)-derived measures are widely used to quantify ITL, as they provide a non-invasive, continuous assessment of cardiovascular load during training.

Advances in wearable technology have enabled practitioners and researchers to objectively quantify ETL. GPS devices with integrated inertial measurement units (IMUs) are now widely used to capture physical demands during training sessions [4,5]. Commonly reported ETL variables include total distance, high-speed running distance, accelerations, decelerations, and player load [6–8]. When combined with HR monitoring, these GPS–IMU-derived variables provide an opportunity to directly examine how specific locomotor and mechanical demands translate into cardiovascular load. Importantly, internal load integrates both exercise intensity and duration, suggesting that accumulated session volume may be a primary driver of cardiovascular strain [9].

Soccer is characterized by repeated accelerations, decelerations, changes of direction, and high-speed running interspersed with periods of low-intensity activity [10–12]. These demands impose substantial mechanical and cardiovascular stress on players, with marked variability both between individuals and within the same players across training sessions [6], even when the external load is identical. Within this framework, the relationship between ETL and ITL represents a key link between prescribed training stimuli and the resulting physiological stress imposed on the athletes. The importance of TL monitoring is further amplified in youth soccer, as adolescent players are exposed to progressively increasing training demands, while undergoing growth- and maturation-related changes in body composition and body size, neuromuscular coordination, and cardiovascular function [13–15]. These developmental processes may further modulate cardiovascular responses to ETL, potentially resulting in greater inter- and intra-individual variability in HR responses compared with adult players [16].

Previous research has examined the associations between GPS-derived ETL variables and HR-derived ITL metrics, demonstrating that cardiovascular responses reflect the cumulative physiological stress imposed by ETL [17]. Volume-based metrics, including total distance and player load, have demonstrated moderate to very large associations with HR-derived measures, whereas intensity variables (e.g., total distance per minute) have shown more variable associations [17,18]. However, many studies have relied on pooled correlation analyses that do not distinguish between between-player and within-player correlations, potentially overestimating associations, when repeated observations are collected across time. Between-player correlations reflect whether changes in one variable are paired with analogous changes in another variable among different individuals. In contrast, within-player correlations describe whether changes in one variable over time are associated with concurrent changes in another variable within the same individual.

Methodological concerns are evident in studies, which include simple correlation analyses, as these may violate the assumptions of independent observations when repeated measures are collected over time and may conflate between-player and within-player effects [19]. Recent research has emphasized the importance of analytical approaches that explicitly account for the longitudinal and clustered structure of TL data, including within-individual correlations, generalized estimating equations, and linear mixed-effects models [18,20,21]. Such approaches allow for the isolation of intra-individual associations between ETL and ITL across training sessions. This provides a more accurate representation of how changes in external load are related to physiological responses within the same player, which is essential for individualizing training prescription and TL monitoring.

Another important consideration is whether ETL should be expressed in volume or in intensity metrics. Volume-based ETL metrics primarily reflect overall training volume, whereas intensity metrics expressed in relation to training time (in minutes) provide insight into training intensity independent of session length [8,22]. While both approaches are widely used in practice, their relative contribution to variability in HR response remains unclear in elite youth populations [23,24]. Given that HR responses are inherently time-dependent, it is less understood whether absolute volume-based metrics or intensity-based variables better explain HR-derived ITL in elite youth soccer training.

Despite the expanding literature on TL monitoring in youth soccer, limited evidence is available on the relationship between absolute and intensity-based GPS metrics using a within-player analytical approach during structured in-season training sessions. Furthermore, few studies have explored the independent predictive contribution of specific ETL variables to HR-based ITL, while accounting for repeated observations within players.

Therefore, the aims of this study were (i) to quantify within-player associations between GPS-derived ETL variables and HR exertion in in-season training sessions in elite under-17 soccer players, and (ii) to determine which intensity-based ETL metrics independently predict HR-based ITL.

## Methods

### Study design

This study was conducted as a retrospective analysis of anonymized routinely collected athlete monitoring data. The data were originally collected as part of regular, non-invasive monitoring procedures in the academy training environment and not specifically for the purposes of this research. The dataset included GPS-derived ETL variables and HR-derived ITL variables collected during regular training sessions across a 9-week in-season period. The anonymized dataset was accessed for research purposes on 7 April 2025. The study was conducted in accordance with the Declaration of Helsinki and was approved by the Institutional Ethics Committee of the Hungarian University of Sports Science for the retrospective research use of anonymized routinely collected athlete monitoring data (MTSE-KEB/No07/2025, 25 March 2025).

### Participants

The anonymized dataset included routinely collected monitoring data from nineteen elite male youth soccer players from an under-17 elite academy team. Players were monitored as part of regular academy practice across nine weeks, from March to May in the 2022/2023 competition season. Only outfield players were included due to the different physical demands of goalkeepers. To ensure sufficient repeated observations for within-player analysis, only players with a minimum of 15 valid monitoring days were included in the final dataset, yielding a total of 266 player-session observations from 11 outfield players (chronological age: 16.4 ± 0.4 years, maturity offset: + 2.5 ± 0.4 years, body height: 177.9 ± 5.3 cm, body mass: 67.7 ± 7.2 kg). Individual observation counts ranged from 16 to 30 sessions per player. Players who did not reach the minimum of 15 valid sessions, mainly due to recurrent short-term illnesses during the monitoring period, were excluded. All participants were free from injury at the time of data collection and took part in regular team training and competitive matches throughout the study. Written informed consent for routine monitoring and the retrospective research use of anonymized monitoring data was obtained from the players’ parents or legal guardians where applicable.

### Anthropometry and maturity assessment

Anthropometric measurements were performed by an ISAK level 2-accredited physiotherapist during the football academy’s routine screening on 12 January 2023. Standing height, sitting height (93.5 ± 3.2 cm), and body mass were recorded, and leg length was calculated as standing height minus sitting height (84.4 ± 3.1 cm). Chronological age was calculated on the date of the anthropometric assessment. Maturity offset, representing the estimated number of years before or after the age at peak height velocity (PHV), was calculated using the equation developed for boys by Mirwald et al. [25]:


$$\begin{array}{cc} \text{Maturity offset }=\;- & \text{9}.\text{236 }+\;(\text{0}.\text{0002708}\times \text{ leg length }\times \text{ sitting height})\;-\;(\text{0}.\text{001663}\times \text{ chronological age }\times \text{ leg length})\;\\ + & \;(\text{0}.\text{007216 }\times \text{ chronological age }\times \text{ sitting height})\;+\;(\text{0}.\text{02292 }\times \text{ body mass}/\text{height ratio}) \end{array}$$

Positive maturity offset values indicate years after PHV, whereas negative values indicate years before the age at PHV.

### External training load monitoring

ETL was quantified using 10-Hz GPS units with integrated 100-Hz IMUs (Catapult S7 Vector, Catapult Sports, Melbourne, Australia). Each player wore the same device throughout the monitoring period to minimize the inter-unit variability. Devices were positioned between the scapulae using a manufacturer-provided sports vest. The tracking unit provides locomotor and mechanical TL variables derived from GPS and tri-axial accelerometer, gyroscope, and magnetometer data. In the present study, the following volume-based ETL variables were used: total distance covered (TDC; m), high-speed running distance (19.8–25.1 km**·**h ^−^ ¹, HSR; m), explosive efforts (EXP; count), and PlayerLoad^TM^ (PL; arbitrary unit, AU). These parameters were also expressed in relation to time, representing intensity-based metrics. HSR was defined according to the manufacturer’s default velocity thresholds. EXP were identified using the proprietary Catapult algorithm based on combined high intensity accelerations and changes of direction (> 3.5 m**·**s ^−^ ²), and deceleration events (<−3.5 m**·**s ^−^ ²). PL was calculated from the vector magnitude of tri-axial accelerometer data and represents a composite measure of the accumulated mechanical TL, according to the manufacturer’s algorithm, originally described by Boyd et al. [26]:


$$\begin{array}{c} PL=\sqrt{\frac{{(a}_{y\text{1}}-a_{y-\text{1}}{)}^{\text{2}}+{(a}_{x\text{1}}-a_{x-\text{1}}{)}^{\text{2}}+{(a}_{z\text{1}}-a_{z-\text{1}}{)}^{\text{2}}}{\text{1}\text{0}\text{0}}}, \end{array}$$

where a represents the acceleration at the y, x, and z axes, respectively.

Previous studies have reported a coefficient of variation (CV) typically below 5% for total distance and player load, whereas higher variability has been observed for high-speed running and accelerations in youth populations (10–15% CV) [1,27–29]. The Catapult Vector unit system has demonstrated acceptable validity for distance-based metrics (typical error ~1–2.5%) and high inter-unit reliability (ICC = 0.95–0.99, 1–3% CV) [30]. In line with recent methodological recommendations for transparent GPS/GNSS reporting in youth soccer proposed by Havanecz et al. [31], key data-quality indicators were reported. GPS signal quality was considered acceptable when the mean number of connected satellites was > 6 and horizontal positioning quality was < 2.0; in the present study, the average satellite count exceeded 6 and mean horizontal dilution of precision (HDOP) remained < 1.0, thereby meeting standardized data quality criteria [29].

### Internal training load monitoring

ITL was assessed via Polar HR sensor (Polar H10, Polar Electro Oy, Kempele, Finland). These devices were attached to a chest strap and worn underneath the GPS vest. The Polar system has demonstrated high validity when compared with electrocardiography and excellent reliability during dynamic exercise conditions [32,33]. Data synchronization was performed with the Catapult OpenField desktop application of the GPS. Individual maximal HR (HRmax) was determined at the beginning of the pre-season by an incremental running test on a treadmill. The test was conducted until the athlete could no longer maintain the required speed, and the highest HR value achieved during the test was considered HRmax. HRmax values were updated manually in the Catapult OpenField Cloud system if higher values were recorded throughout the season. ITL was expressed as heart rate exertion (HR exertion, AU), calculated using a HR-zone-based training impulse approach, which considers the time spent in individualized HR zones relative to HRmax, and is expressed as a cumulative weighted measure, which is provided by the manufacturer’s default settings. HR exertion was used as a cumulative HR-zone-based indicator of cardiovascular training load [17]. This metric is related to established TRIMP-based approaches, as it integrates exercise intensity and duration into a single weighted score. The following calculation was used to describe HR exertion with eight HR zones: time spent in zone 1 (≤ 45% of HRmax) and multiplied by 1, time spent in zone 2 (45–55% of HRmax) and multiplied by 1.122, time spent in zone 3 (55–65% of HRmax) and multiplied by 1.322, time spent in zone 4 (65–75% of HRmax) and multiplied by 1.554, time spent in zone 5 (75–85% of HRmax) and multiplied by 2.037, time spent in zone 6 (85–95% of HRmax) and multiplied by 3.252, time spent in zone 7 (95–105% of HRmax) and multiplied by 5.439, and time spent in zone 8 (> 105% of HRmax) and multiplied by 9.0. These scores were summarized and expressed in AU. Zones 7 and 8 bands were software-defined relative to the reference HRmax stored in OpenField. Therefore, HR values above 100% indicate that the recorded HR exceeded the stored reference value, which may not fully reflect the athlete’s current HRmax. The HR-zone method integrates exercise intensity and duration into a single composite score and is conceptually related to established TRIMP-based models proposed in well-known previous studies [34-36].

### Training sessions

Training sessions were structured according to a typical weekly microcycle relative to match day (MD) [37]. A microcycle consisted of five training days (Monday to Friday), with each training day having a distinct training goal. Monday (MD + 2/MD-5) sessions primarily focused on facilitating physiological recovery, while maintaining technical engagement. These sessions typically lasted 45–60 mins and included moderate-intensity aerobic activities, technical ball work at reduced intensity (compared to any other training day), and small-sided possession games. Work-to-rest ratios were generally of low-demand, with frequent passive recovery periods (1:1 or greater). Tuesday (MD + 3/MD-4) sessions emphasized agility and neuromuscular coordination development. Training duration ranged from 60 to 75 mins and included change-of-direction drills, short acceleration tasks (typical range between 5–20 m), agility exercises, and small- to medium-sided games. Exercises were organized in short bouts (5–15 s) with moderate-to-long recovery intervals (1:3–1:5) to maintain movement quality. Wednesday (MD-3) represented the highest volume-based TL. Sessions lasted approximately 75–90 mins and were centered on large-area games and tactical exercises designed to replicate match-like physical demands for all players. Large-sided games (8v8–11v11), transition drills, and extended tactical sequences were implemented in these sessions. Work periods were typically longer (2–6 mins), interspersed with structured recovery phases (1–3 mins), resulting in elevated locomotor and cardiovascular demands. Thursday (MD-2) sessions focused on speed and high-intensity actions. Training duration was approximately 60–75 mins and included maximal sprint efforts (10–30 m), repeated sprint drills, and position-specific speed exercises, often integrated into tactical scenarios. Efforts were brief (≤ 10 s) with long recovery intervals (1:4–1:6) to ensure high movement quality and maximal velocity exposure. Friday (MD-1) sessions served as neuromuscular activation training before the weekend match. These sessions were shorter in duration (45–60 mins) and consisted of low-volume, moderate- to high-intensity technical and tactical tasks, short accelerations, and relay race-type agility games. Work bouts were brief, with ample recovery to avoid substantial fatigue.

### Statistical analysis

Descriptive statistics are presented as mean ± SD, including minimum and maximum values. Within-player associations between ETL variables and HR exertion were examined using repeated measures correlation, which accounts for the non-independence of repeated observations within players [38]. Analyses were performed in R (version 4.6.1, R Foundation for Statistical Computing, Vienna, Austria) using the rmcorr package (version 0.7.0). All training sessions were retained as individual observations. This method estimates a common within-subject slope by removing between-subject variability through subject-mean centering, thereby isolating intra-individual associations across training sessions. Correlation coefficients (r), 95% confidence intervals (CI), and associated p-values were reported, with statistical significance set at α = 0.05. To examine the predictive contribution of intensity-based ETL variables to HR exertion·min ^−^ ¹, a linear mixed-effects model was constructed using JASP (version 0.95.4, JASP Team, Amsterdam, Netherlands). Player identity (numerical order) was included as a random intercept, while TDC·min ^−^ ¹, HSR·min ^−^ ¹, EXP·min ^−^ ¹, and maturity offset were entered as fixed effects. A sensitivity model was also fitted with playing position included as an additional fixed effect. Multicollinearity was examined using variance inflation factors (VIF), with all values being below 2.0, indicating no concern. Model assumptions were checked by visual inspection of residual plots and Q–Q plots. Potential influential observations were examined using standardized residuals and Cook’s distance. Effect magnitudes were interpreted as trivial (< 0.1), small (0.1–0.29), moderate (0.30–0.49), large (0.50–0.69), very large (0.70–0.89), and nearly perfect (≥ 0.90) [39].

## Results

Descriptive statistics for the examined variables are presented in Table 1.

**Table 1:** Descriptive statistics for the examined external training and internal training load variables.

Variables	Mean	SD	Min	Max
HR exertion (AU)	4695.1	1370.0	2255.5	9096.3
TDU (min)	50.0	11.8	30.1	78.4
TDC (m)	4144.9	1401.9	1966.7	8478.2
HSR (m)	130.5	97.8	0.0	892.1
EXP (count)	18.1	8.9	1.0	48.0
PL (AU)	460.9	163.6	203.6	937.5
TDC·min ^−^ ¹ (m)	81.8	13.1	50.8	116.5
HSR·min ^−^ ¹ (m)	2.5	1.5	0.0	12.5
EXP·min ^−^ ¹ (count)	0.4	0.1	0.1	0.8
PL·min ^−^ ¹ (AU)	9.1	1.9	5.5	13.7

Abbreviations: HR exertion: heart rate exertion; AU: arbitrary unit; TDU: training duration; TDC: total distance covered; HSR: high-speed running distance; EXP: explosive efforts; PL: PlayerLoad^TM^

**Fig 1:**
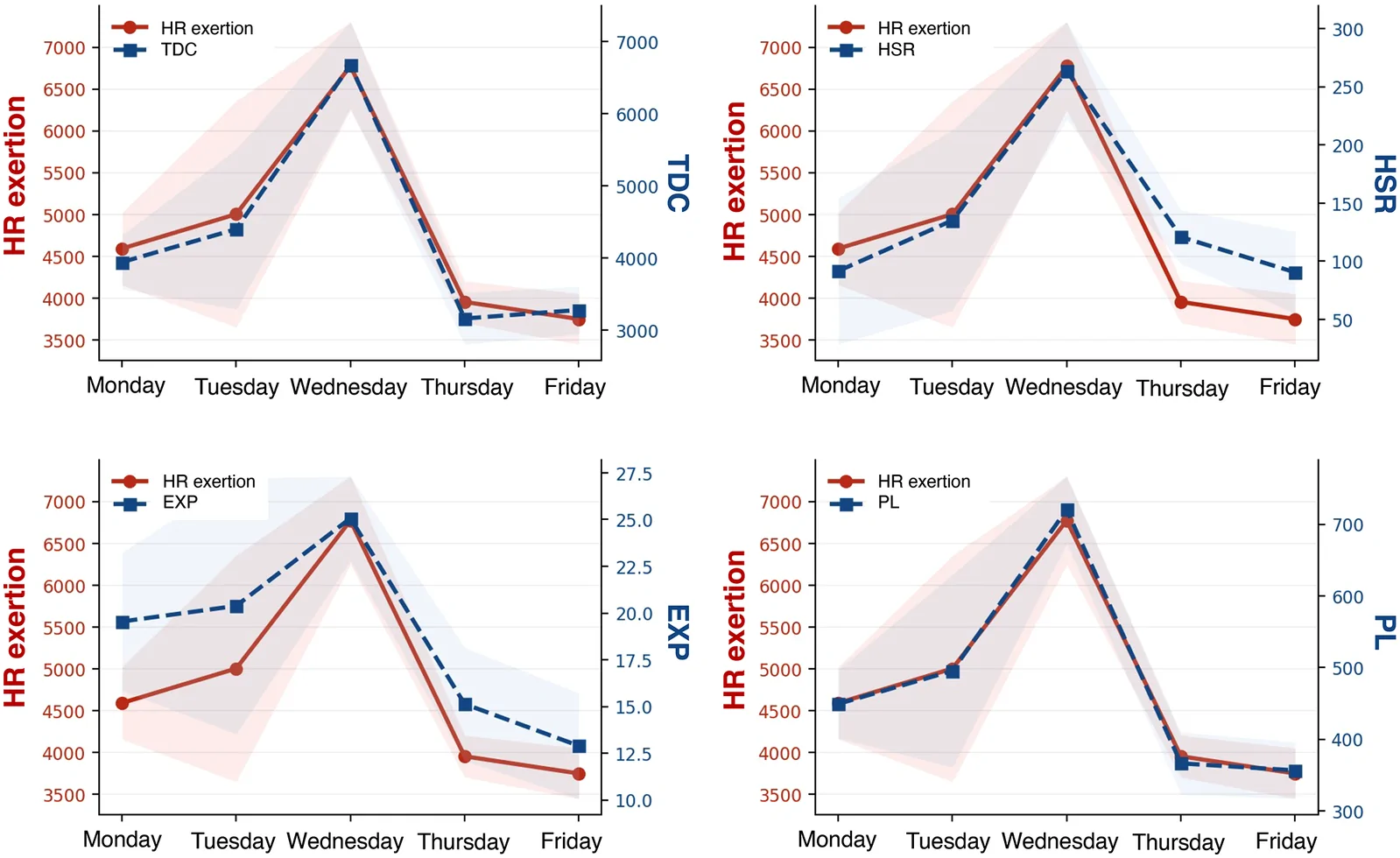
Daily variation in HR exertion and volume-based ETL variables within a microcycle. TDC: total distance covered, HSR: high-speed running distance, EXP: explosive efforts, PL: PlayerLoad^TM^.

**Fig 2:**
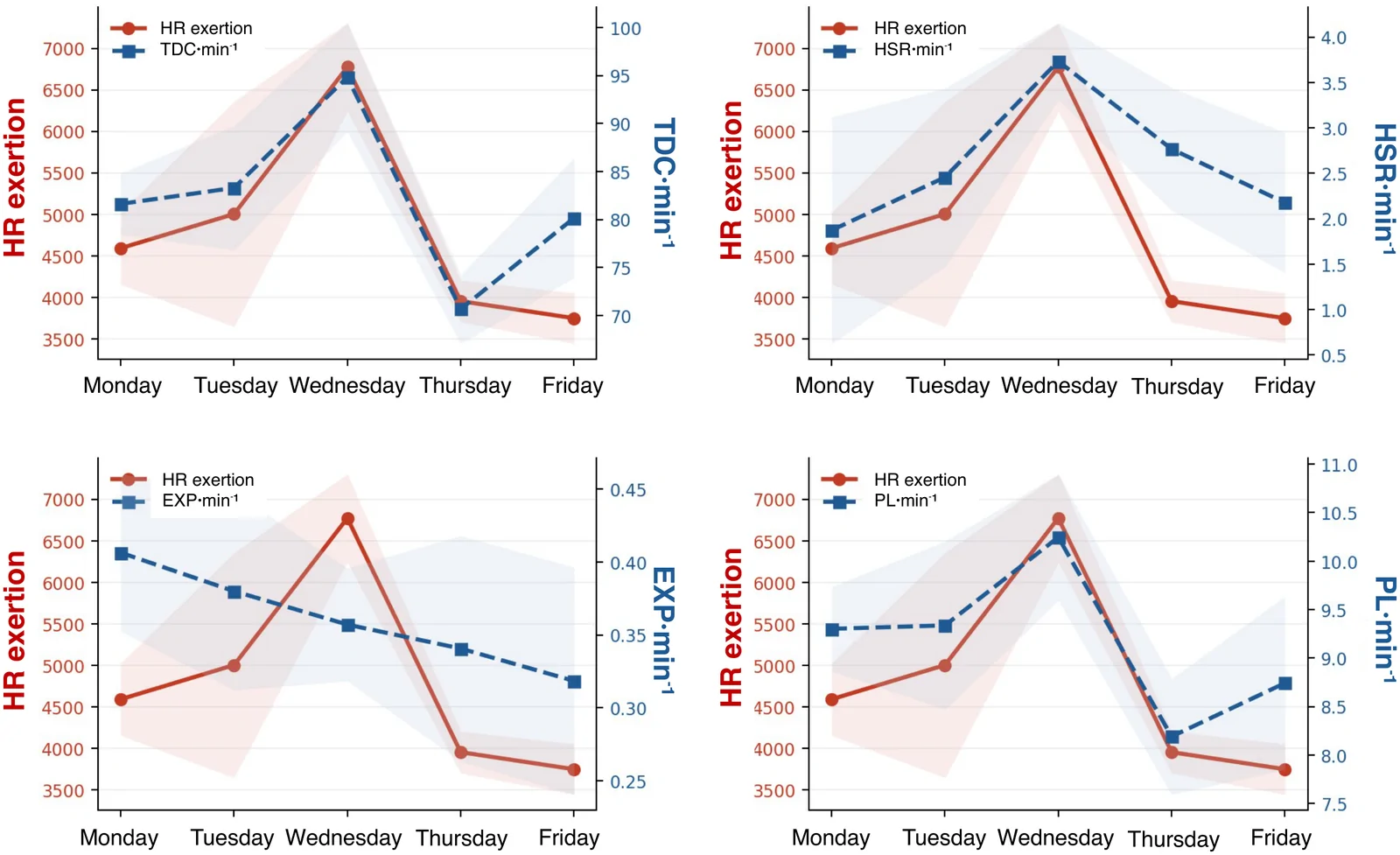
Daily variation in HR exertion and intensity-based ETL variables within a microcycle. TDC: total distance covered, HSR: high-speed running distance, EXP: explosive efforts, PL: PlayerLoad^TM^.

As illustrated in Figs 1 and 2, HR exertion and ETL variables demonstrated similar temporal patterns across training days, with peak values observed mid-week (Wednesday) and lower values toward the end of the training week (Friday). Notably, this alignment was more pronounced for volume-based ETL variables (Fig 1) compared to intensity-based variables (Fig 2).

Repeated measures correlation revealed nearly perfect within-player associations between HR exertion and both TDC (r = 0.921, 95% CI: 0.903–0.946, p < 0.001) and PL (r = 0.924, 95% CI: 0.906–0.948, p < 0.001). HSR (r = 0.662, 95% CI: 0.587–0.726, p < 0.001) and EXP (r = 0.621, 95% CI: 0.542–0.690, p < 0.001) demonstrated large associations with HR exertion. Intensity metrics showed smaller effect sizes compared to absolute variables. Large correlations were observed for TDC·min ^−^ ¹ (r = 0.543) and PL·min ^−^ ¹ (r = 0.503), and a moderate correlation was observed for HSR·min ^−^ ¹ (r = 0.390), whereas EXP·min ^−^ ¹ (r = 0.195) demonstrated a small association (p = 0.002).

The linear mixed-effects model predicting HR exertion·min ^−^ ¹ showed that TDC·min ^−^ ¹ demonstrated the strongest independent effect, followed by EXP·min ^−^ ¹. In contrast, HSR·min ^−^ ¹ and maturity offset were not independently associated with HR exertion·min ^−^ ¹ (Table 2). Multicollinearity diagnostics indicated no evidence of collinearity among the predictors (VIF = 1.087–1.121).

**Table 2:** Linear mixed-effects model predicting HR exertion·min ^−^ ¹ by intensity-based ETL variables and maturity offset.

Predictor	β	SE	df	t	p
Intercept	28.400	10.890	11.770	2.609	0.023
TDC·min ⁻ ¹	0.490	0.052	246.300	9.388	<0.001
HSR·min ⁻ ¹	0.390	0.371	256.000	1.051	0.294
EXP·min ⁻ ¹	21.870	3.929	257.500	5.567	<0.001
Maturity offset	6.344	3.829	8.185	1.657	0.135

Abbreviations: HR exertion·min ^−^ ¹: heart rate exertion per minute; TDC·min ^−^ ¹: total distance covered per minute (m); HSR·min ^−^ ¹: high-speed running distance per minute (19.8–25.1 km**·**h ^−^ ¹; m); EXP·min ^−^ ¹: explosive efforts per minute (> 3.5 m**·**s ^−^ ² acceleration/change-of-direction or <−3.5 m**·**s ^−^ ² deceleration events). Note: Player ID was included as a random intercept (variance of 24.74, residual variance: 67.17, AIC: 1901, BIC: 1926). The model was fitted using restricted maximum likelihood and Satterthwaite’s method was used for testing model terms.

Including playing position in the model did not alter the overall pattern of associations. TDC·min ^−^ ¹ (β = 0.482, p < 0.001) and EXP·min ^−^ ¹ (β = 21.920, p < 0.001) remained significant predictors, whereas HSR·min ^−^ ¹ (p = 0.265) and maturity offset (p = 0.739) remained non-significant. No statistically significant position-related differences were observed.

## Discussion

The present study examined within-player associations between GPS-derived ETL variables and HR-based ITL during in-season training in elite youth soccer players. The main finding was that HR exertion was most strongly associated with TDC and PL. In contrast, TDC·min ^−^ ¹ and EXP·min ^−^ ¹ were the main independent predictors of HR exertion·min ^−^ ¹, whereas HSR·min ^−^ ¹ did not provide additional explanatory value. These findings indicate that HR-based load metrics reflect different aspects of the external–internal training load relationship.

The daily patterns provide additional insights for these associations (Figs 1 and 2). HR exertion and volume-based ETL variables followed a broadly similar weekly pattern, with the highest values observed around the mid-week training day. This is consistent with the structure of the weekly microcycle, in which MD-3 sessions typically represented the highest-volume stimulus. In contrast, the results showed greater variability in the external–internal load relationship, especially on Monday and Tuesday, which may reflect the more heterogeneous training aims on these days, including technical activities, agility tasks, and individualized load management following the preceding match. In these training days, increases in, particularly, EXP were not paired with an analogous increase in HR exertion. Similar day-to-day variation in TL across soccer microcycles has been reported previously, when training content differs according to match-day proximity and age category-specific programming [40]. These descriptive patterns should not be interpreted as evidence of day-specific external–internal load relationships, as training day was not formally included as a predictor in the analysis.

The nearly perfect within-player associations between HR exertion and both TDC and PL indicate that accumulated cardiovascular load closely reflected the volume of external work performed within individual players. However, this finding should be interpreted in light of the cumulative nature of HR exertion. Because HR exertion integrates both exercise intensity and duration, strong associations with volume-based ETL variables are partly expected. Therefore, the main practical value of the present findings is not merely the strength of these associations, but the distinction between cumulative and time-normalized interpretations of internal load. In this context, HR exertion appears more suitable for evaluating accumulated cardiovascular load across the full training session, whereas HR exertion·min ^−^ ¹ provides a more specific indication of per-minute internal load intensity. Accordingly, greater total distance and greater mechanical load are likely to be accompanied by higher cardiovascular strain across training sessions. Previous studies have also demonstrated strong relationships between accumulated external load and cardiovascular responses in team sports [7,18]. In addition, several studies have also demonstrated the concept that HR-derived internal load reflects the cumulative physiological stress imposed by exercise, which is mainly driven by the total volume of work performed [3,9]. In soccer-specific research, TDC and PL have been identified as key load variables, integrating a wide range of movement-specific activities performed during training [6,7], which may explain their strong association with HR exertion in the present within-player analysis.

When HR exertion was expressed relative to training duration, TDC·min ^−^ ¹ emerged as the strongest independent predictor of HR exertion·min ^−^ ¹. This suggests that the overall locomotor intensity of training remained closely associated with per-minute cardiovascular strain. This interpretation is consistent with the concept that HR-derived load reflects the physiological response to the external work performed, but that the strength and meaning of this relationship depends on whether load is expressed in absolute or relative terms [3,9,18]. EXP·min ^−^ ¹ also contributed independently, suggesting that explosive actions may provide additional information about the intensity of the internal response. This may be relevant because acceleration-, deceleration-, and change-of-direction-based actions impose distinct mechanical and physiological demands that are not fully captured by distance-based variables alone [41]. In contrast, HSR·min ^−^ ¹ was not independently associated with HR exertion·min ^−^ ¹ after accounting for TDC·min ^−^ ¹ and EXP·min ^−^ ¹, suggesting that high-speed running intensity did not provide additional explanatory value in this training context.

An important methodological aspect of the present study is the use of a within-player analytical approach to examine the relationship between ETL and ITL. Previous studies have often relied on pooled or group-level analyses, reporting associations between external and internal load without separating between- and within-player effects [7,17,42,43]. While these studies consistently demonstrate moderate-to-very large relationships between ETL and ITL variables, such approaches may be influenced by inter-individual variation and may therefore not reflect intra-individual changes across repeated training sessions [19]. By accounting for repeated observations within players, repeated-measures correlation provides a more direct estimate of how fluctuations in ETL are accompanied by changes in ITL within the same player [38]. This is relevant for training monitoring, where practitioners are often interested in whether an individual player shows a higher or lower internal response than expected for a given external training stimulus.

Regarding youth physical development, adolescent soccer players are exposed to ongoing growth- and maturation-related changes that influence physiological responses to TL [14,16]. These developmental factors may contribute to greater inter- and intra-individual variability in HR responses compared with adult athletes [18]. In previous research with sub-elite youth soccer players [44] the strength of the correlations progressively decreased with the decrease in age, with the lowest associations being found in the age group close to the age at peak height velocity (under-15). In our study, maturity offset was not associated with HR exertion·min ^−^ ¹. This may partly reflect the relatively narrow post-PHV range, as most athletes were from two to three years after their age at PHV, and contradicts previous findings reporting maturity-related differences in training load across age and maturity groups in youth soccer [45]. The present within-player findings suggest that changes in external load were closely reflected by changes in HR data within individual players, especially for volume-based variables. These strong correlations also indicate that in the under-17 age group, the associations between ETL and ITL are of similar magnitude to that observed for adult players. This further highlights the importance of individualized monitoring of training load in youth soccer, which may contribute to more individualized load management and training prescription.

Based on the present findings, the use of solely external metrics (e.g., total distance, high-speed running distance) may overlook individual variability in physiological adaptation. From a practical point of view, the HR exertion appears useful for evaluating the cardiovascular cost of a training session, in relation to volume-based GPS variables such as TDC and PL. In contrast, HR exertion·min ^−^ ¹ may provide additional insight into the intensity of the internal response, with TDC·min ^−^ ¹ and EXP·min ^−^ ¹ being the most relevant ETL indicators in the present model. Therefore, combining ETL and ITL metrics, alongside appropriate within-player analytical approaches, may provide more precise TL monitoring and support further decision-making in training prescription.

### Limitations

This study has several limitations. The sample included players from a single elite youth soccer academy team and was collected during a specific period of the competitive season, which limits the generalizability of the findings to other age groups, competitive levels, and training environments. Although the dataset included 266 player-session observations, these observations were nested within only 11 players; therefore, the findings should be interpreted as exploratory and context-specific. The small number of players within each positional category also limited robust inference regarding playing position differences. Although maturity offset was included as a player-level covariate, the small number of players and the relatively narrow maturity range limited the assessment of maturation-related differences. Only training sessions were included, and the external–internal load relationships observed may not generalize to match play. ITL was quantified using a manufacturer-derived HR exertion metric, which reflects cardiovascular strain but does not capture perceptual, neuromuscular, or psychological aspects of internal load. Finally, the EXP variable was derived from a proprietary algorithm, which may limit comparability with studies using different tracking systems, thresholds, or event-detection methods.

### Practical implications

Practitioners should avoid interpreting HR exertion and HR exertion·min ^−^ ¹ as interchangeable indicators. HR exertion may be more appropriate for evaluating accumulated cardiovascular load across a full training session, in relation to volume-based GPS variables such as TDC and PL. In contrast, HR exertion·min ^−^ ¹ may provide more specific information about the intensity of the internal response, especially when interpreted alongside TDC·min ^−^ ¹ and EXP·min ^−^ ¹. This distinction may help coaches identify whether a player’s cardiovascular response reflects greater total training volume or a higher training intensity. Accordingly, combining these variables may support more individualized interpretation of training responses in elite youth soccer.

## Conclusion

In elite youth soccer training, HR exertion showed very strong within-player associations with TDC and PL. TDC·min ^−^ ¹ and EXP·min ⁻ ¹ emerged as the main independent predictors of HR exertion·min ^−^ ¹. These findings indicate that cumulative and time-normalized HR-based load metrics should be interpreted separately, as they reflect different aspects of the external–internal load relationship. Within-player analytical approaches may therefore provide a more individualized interpretation of training responses in elite under-17 youth soccer.

## Abbreviations

AU: arbitrary unit
CI: confidence interval
CV: coefficients of variation
ETL: external training load
EXP: explosive efforts
GPS: global positioning system
HR: heart rate
HRmax: maximal heart rate
HSR: high-speed running distance
IMU: inertial measurement unit
ICC: intraclass correlation coefficient
ITL: internal training load
MD: match day
PL: PlayerLoad^TM^
TDC: total distance covered
TDU: total duration
TL: training load

## References

[1] Varley MC, Fairweather IH, Aughey RJ. Validity and reliability of GPS for measuring instantaneous velocity during acceleration, deceleration, and constant motion. J Sports Sci. 2012;30(2):121–7. doi: 10.1080/02640414.2011.627941 22122431

[2] Bourdon PC, Cardinale M, Murray A, Gastin P, Kellmann M, Varley MC, et al. Monitoring Athlete Training Loads: Consensus Statement. Int J Sports Physiol Perform. 2017;12(Suppl 2):S2161–70. doi: 10.1123/IJSPP.2017-0208 28463642

[3] Impellizzeri FM, Rampinini E, Marcora SM. Physiological assessment of aerobic training in soccer. J Sports Sci. 2005;23(6):583–92. doi: 10.1080/02640410400021278 16195007

[4] Cummins C, Orr R, O’Connor H, West C. Global positioning systems (GPS) and microtechnology sensors in team sports: a systematic review. Sports Med. 2013;43(10):1025–42. doi: 10.1007/s40279-013-0069-2 23812857

[5] Scott MTU, Scott TJ, Kelly VG. The Validity and Reliability of Global Positioning Systems in Team Sport: A Brief Review. J Strength Cond Res. 2016;30(5):1470–90. doi: 10.1519/JSC.0000000000001221 26439776

[6] Akenhead R, Nassis GP. Training Load and Player Monitoring in High-Level Football: Current Practice and Perceptions. Int J Sports Physiol Perform. 2016;11(5):587–93. doi: 10.1123/ijspp.2015-0331 26456711

[7] Barrett S, Midgley A, Lovell R. PlayerLoad™: reliability, convergent validity, and influence of unit position during treadmill running. Int J Sports Physiol Perform. 2014;9(6):945–52. doi: 10.1123/ijspp.2013-0418 24622625

[8] Delaney JA, Duthie GM, Thornton HR, Scott TJ, Gay D, Dascombe BJ. Acceleration-Based Running Intensities of Professional Rugby League Match Play. Int J Sports Physiol Perform. 2016;11(6):802–9. doi: 10.1123/ijspp.2015-0424 26693738

[9] Halson SL. Monitoring training load to understand fatigue in athletes. Sports Med. 2014;44(Suppl 2):S139-47. doi: 10.1007/s40279-014-0253-z 25200666 PMC4213373

[10] Di Salvo V, Baron R, Tschan H, Calderon Montero FJ, Bachl N, Pigozzi F. Performance characteristics according to playing position in elite soccer. Int J Sports Med. 2007;28(3):222–7. doi: 10.1055/s-2006-924294 17024626

[11] Mohr M, Krustrup P, Bangsbo J. Match performance of high-standard soccer players with special reference to development of fatigue. J Sports Sci. 2003;21(7):519–28. doi: 10.1080/0264041031000071182 12848386

[12] Reilly T. Energetics of high-intensity exercise (soccer) with particular reference to fatigue. J Sports Sci. 1997;15(3):257–63. doi: 10.1080/026404197367263 9232551

[13] Lloyd RS, Cronin JB, Faigenbaum AD, Haff GG, Howard R, Kraemer WJ, et al. National Strength and Conditioning Association Position Statement on Long-Term Athletic Development. J Strength Cond Res. 2016;30(6):1491–509. doi: 10.1519/JSC.0000000000001387 26933920

[14] Malina RM, Eisenmann JC, Cumming SP, Ribeiro B, Aroso J. Maturity-associated variation in the growth and functional capacities of youth football (soccer) players 13-15 years. Eur J Appl Physiol. 2004;91(5–6):555–62. doi: 10.1007/s00421-003-0995-z 14648128

[15] Meylan C, Trewin J, McKean K. Quantifying Explosive Actions in International Women’s Soccer. Int J Sports Physiol Perform. 2017;12(3):310–5. doi: 10.1123/ijspp.2015-0520 27295719

[16] Buchheit M, Mendez-Villanueva A. Effects of age, maturity and body dimensions on match running performance in highly trained under-15 soccer players. J Sports Sci. 2014;32(13):1271–8. doi: 10.1080/02640414.2014.884721 24786981

[17] Havanecz K, Sáfár S, Bartha C, Kopper B, Horváth T, Tóth PJ, et al. Relationship Between Internal and External Load in Under-16 Soccer Players: Heart Rate, Rating of Perceived Exertion, and GPS-Derived Variables. Sports (Basel). 2025;13(11):376. doi: 10.3390/sports13110376 41295759 PMC12656065

[18] McLaren SJ, Macpherson TW, Coutts AJ, Hurst C, Spears IR, Weston M. The Relationships Between Internal and External Measures of Training Load and Intensity in Team Sports: A Meta-Analysis. Sports Med. 2018;48(3):641–58. doi: 10.1007/s40279-017-0830-z 29288436

[19] Stevens TGA, de Ruiter CJ, Twisk JWR, Savelsbergh GJP, Beek PJ. Quantification of in-season training load relative to match load in professional Dutch Eredivisie football players. Science and Medicine in Football. 2017;1(2):117–25. doi: 10.1080/24733938.2017.1282163

[20] Marynowicz J, Kikut K, Lango M, Horna D, Andrzejewski M. Relationship Between the Session-RPE and External Measures of Training Load in Youth Soccer Training. J Strength Cond Res. 2020;34(10):2800–4. doi: 10.1519/JSC.0000000000003785 32773542

[21] Twisk JW. Applied longitudinal data analysis for epidemiology: a practical guide. Cambridge: Cambridge University Press. 2013. doi: 10.1017/CBO9781139342834

[22] Jaspers A, Brink MS, Probst SGM, Frencken WGP, Helsen WF. Relationships Between Training Load Indicators and Training Outcomes in Professional Soccer. Sports Med. 2017;47(3):533–44. doi: 10.1007/s40279-016-0591-0 27459866

[23] Miguel M, Oliveira R, Loureiro N, García-Rubio J, Ibáñez SJ. Load Measures in Training/Match Monitoring in Soccer: A Systematic Review. Int J Environ Res Public Health. 2021;18(5):2721. doi: 10.3390/ijerph18052721 33800275 PMC7967450

[24] Oliveira R, Brito JP, Martins A, Mendes B, Marinho DA, Ferraz R, et al. In-season internal and external training load quantification of an elite European soccer team. PLoS One. 2019;14(4):e0209393. doi: 10.1371/journal.pone.0209393 31009464 PMC6476476

[25] Mirwald RL, Baxter-Jones ADG, Bailey DA, Beunen GP. An assessment of maturity from anthropometric measurements. Med Sci Sports Exerc. 2002;34(4):689–94. doi: 10.1097/00005768-200204000-00020 11932580

[26] Boyd LJ, Ball K, Aughey RJ. The reliability of MinimaxX accelerometers for measuring physical activity in Australian football. Int J Sports Physiol Perform. 2011;6(3):311–21. doi: 10.1123/ijspp.6.3.311 21911857

[27] Fox JL, Stanton R, Sargent C, Wintour S-A, Scanlan AT. The Association Between Training Load and Performance in Team Sports: A Systematic Review. Sports Med. 2018;48(12):2743–74. doi: 10.1007/s40279-018-0982-5 30225537

[28] Johnston RJ, Watsford ML, Kelly SJ, Pine MJ, Spurrs RW. Validity and interunit reliability of 10 Hz and 15 Hz GPS units for assessing athlete movement demands. J Strength Cond Res. 2014;28(6):1649–55. doi: 10.1519/JSC.0000000000000323 24276300

[29] Malone JJ, Lovell R, Varley MC, Coutts AJ. Unpacking the black box: applications and considerations for using GPS devices in sport. Int J Sports Physiol Perform. 2017;12(Suppl 2):S2-18–S2-26. doi: 10.1123/ijspp.2016-0236 27736244

[30] Thornton HR, Nelson AR, Delaney JA, Serpiello FR, Duthie GM. Interunit Reliability and Effect of Data-Processing Methods of Global Positioning Systems. Int J Sports Physiol Perform. 2019 Apr 1;14(4):432–8. doi: 10.1123/ijspp.2018-0273 30204529

[31] Havanecz K, Matlák J, Ihász F, Géczi G, Kopper B, Sáfár S, et al. A Call for Consensus: A Narrative Review of GPS-Based External Training Load Monitoring in Male Youth Soccer Players. Sports (Basel). 2026;14(4):152. doi: 10.3390/sports14040152 42043084 PMC13119910

[32] Giles D, Draper N, Neil W. Validity of the Polar V800 heart rate monitor to measure RR intervals at rest. Eur J Appl Physiol. 2016;116(3):563–71. doi: 10.1007/s00421-015-3303-9 26708360 PMC4751190

[33] Gilgen-Ammann R, Schweizer T, Wyss T. RR interval signal quality of a heart rate monitor and an ECG Holter at rest and during exercise. Eur J Appl Physiol. 2019;119(7):1525–32. doi: 10.1007/s00421-019-04142-5 31004219

[34] Edwards S. The Heart Rate Monitor Book. Medicine & Science in sports & Exercise. 1994;26(5):647. doi: 10.1249/00005768-199405000-00020

[35] Lucia A, Hoyos J, Santalla A, Earnest C, Chicharro JL. Tour de France versus Vuelta a España: which is harder? Med Sci Sports Exerc. 2003;35(5):872–8. doi: 10.1249/01.MSS.0000064999.82036.B4 12750600

[36] Banister EW. Modeling elite athletic performance. Physiological testing of elite athletes. Champaign: Human Kin. 1991. p. 403–22.

[37] Bompa TO, Buzzichelli CA. Periodization: theory and methodology of training. 6th ed. Champaign (IL): Human Kinetics; 2019.

[38] Bakdash JZ, Marusich LR. Repeated Measures Correlation. Front Psychol. 2017;8:456. doi: 10.3389/fpsyg.2017.00456 28439244 PMC5383908

[39] Hopkins WG, Marshall SW, Batterham AM, Hanin J. Progressive Statistics for Studies in Sports Medicine and Exercise Science. Medicine & Science in Sports & Exercise. 2009;41(1):3–13. doi: 10.1249/MSS.0b013e31818cb278 19092709

[40] Franceschi A, Robinson MA, Owens DJ, Brownlee T, Bampouras TM, Ferrari Bravo D, et al. Training loads and microcycle periodisation in Italian Serie A youth soccer players. J Sports Sci. 2024;42(15):1410–20. doi: 10.1080/02640414.2024.2391648 39172819

[41] Vanrenterghem J, Nedergaard NJ, Robinson MA, Drust B. Training Load Monitoring in Team Sports: A Novel Framework Separating Physiological and Biomechanical Load-Adaptation Pathways. Sports Med. 2017;47(11):2135–42. doi: 10.1007/s40279-017-0714-2 28283992

[42] Casamichana D, Castellano J, Calleja-Gonzalez J, San Román J, Castagna C. Relationship between indicators of training load in soccer players. J Strength Cond Res. 2013;27(2):369–74. doi: 10.1519/JSC.0b013e3182548af1 22465992

[43] Scott BR, Lockie RG, Knight TJ, Clark AC, Janse de Jonge XAK. A comparison of methods to quantify the in-season training load of professional soccer players. Int J Sports Physiol Perform. 2013;8(2):195–202. doi: 10.1123/ijspp.8.2.195 23428492

[44] Teixeira JE, Forte P, Ferraz R, Leal M, Ribeiro J, Silva AJ, et al. The Association between External Training Load, Perceived Exertion and Total Quality Recovery in Sub-Elite Youth Football. TOSSJ. 2022;15(1):e1875399X2207220. doi: 10.2174/1875399x-v15-e2207220

[45] Teixeira JE, Alves AR, Ferraz R, Forte P, Leal M, Ribeiro J, et al. Effects of Chronological Age, Relative Age, and Maturation Status on Accumulated Training Load and Perceived Exertion in Young Sub-Elite Football Players. Front Physiol. 2022;13:832202. doi: 10.3389/fphys.2022.832202 35432006 PMC9010324

